# The COVID-19 pandemic and social cognitive outcomes in early childhood

**DOI:** 10.1038/s41598-024-80532-w

**Published:** 2024-11-22

**Authors:** Rose M. Scott, Gabriel Nguyentran, James Z. Sullivan

**Affiliations:** 1 Psychological Sciences, School of Social Sciences, Humanities, and Arts, University of California Merced, 5200 North Lake Road, Merced, CA 95343 USA; 2 Health Sciences Research Institute, University of California Merced, 5200 North Lake Road, Merced, CA 95343 USA

**Keywords:** COVID-19, Child development, Cognitive development, Social cognition, False-belief understanding, Socioeconomic status, Psychology, Human behaviour

## Abstract

The COVID-19 pandemic and ensuing lockdowns led to sweeping changes in the everyday lives of children and families, including school closures, remote work and learning, and social distancing. To date no study has examined whether these profound changes in young children’s day to day social interactions impacted the development of social cognition skills in early childhood. To address this question, we compared the performance of two cohorts of 3.5- to 5.5-year-old children tested before and after the COVID-19 lockdowns on several measures of false-belief understanding, a critical social cognition skill that undergoes important developments in this age range. Controlling for age and language skills, children tested after the pandemic demonstrated significantly worse false-belief understanding than those tested before the pandemic, and this difference was larger for children from lower socioeconomic status (SES) backgrounds. These results suggest that the pandemic negatively impacted the development of social cognition skills in early childhood, especially for lower SES children.

**Supplementary Information:**

The online version contains supplementary material available at 10.1038/s41598-024-80532-w.

The COVID-19 pandemic caused considerable disruption in the lives of children and families, as governments issued shelter-in-place orders and encouraged social distancing, schools and daycares closed, and many transitioned to remote work and online learning. It is critical to understand how these unprecedented circumstances impacted child development.

Much of the research on this issue has focused on school-aged children^1–6^. This work has identified negative impacts on children’s mental health and emotional well-being, including increases in depression, anxiety, and behavioral problems, as well as increases in family conflict and harsh parenting^4–9^. Other studies have identified delays in children’s learning across a variety of domains^1–3^. In a recent meta-analysis of 42 studies, Betthäuser et al.^1^ estimated that, averaged across all grades and subjects, children lost 35% of a school year’s worth of learning. This loss remained stable over time, despite returns to in-person schooling. They also found that the pandemic exaggerated social inequalities, with children from lower socioeconomic status (SES) backgrounds suffering greater learning losses^1^.

Relatively less work has focused on the potential impacts of the pandemic on younger children. However, some studies suggest that the pandemic also had negative impacts prior to the school years^10–14^. For instance, Sato et al.^14^ found that 3- to 5-year-old children in Japan exhibited delays in overall development relative to pre-pandemic cohorts. González et al.^11^ similarly found that preschool closures in Uruguay were linked to losses in school-readiness in 4- and 5-year-old children and these losses were greater for children from lower SES households. Other studies suggest that pandemic lockdowns were associated with increases in screen time for children^15^, including young infants^10,12,13^. These increases in screen time were larger in locations that experienced longer lockdowns and for families who experienced a loss of childcare^10,15^. Increased screen time during lockdown has in turn been linked with slower vocabulary development for infants and toddlers during the pandemic^10,12^. However, Kartushina et al.^12^ found that infants and toddlers in higher SES families showed accelerated gains in vocabulary during lockdown, and these gains were associated with shared book reading with parents. It is possible that for some higher SES families, lockdowns may have created increased opportunities for one-on-one parent-child interactions with positive effects on developmental outcomes. Thus, the impact of lockdowns on early childhood may differ across families and socioeconomic strata.

One area that has received insufficient attention is potential impacts of the pandemic on the development of social cognition skills in early childhood. Here we focused on the development of a specific social cognition skill, the ability to recognize that other individuals can be mistaken or hold false beliefs about the world. False-belief understanding requires the ability to distinguish between the mind and reality – to recognize that mental states (e.g., goals, feelings, beliefs) are subjective internal representations that can differ across individuals and be wrong. This important skill is thought to play a vital role in cooperation, communication, and learning^16,17^ and it is associated with a broad range of positive developmental outcomes including social competence, prosocial behavior, and academic achievement^18–20^. The development of false-belief understanding has thus been the focus of considerable research for several decades.

Although there is ongoing debate about when children begin to represent others’ beliefs, it is widely agreed that children’s ability to engage in successful false-belief reasoning undergoes important developments in the first five years of life^21–26^. False-belief reasoning skills during this period have been linked to a variety of social factors^27^, including the quantity and quality of children’s social interactions with parents, siblings, and peers^28–30^. Thus, shifts in the frequency and nature of social interactions during the pandemic, together with increases in alternative activities such as passive screen time, could potentially have negatively impacted the development of false-belief understanding in early childhood. Moreover, these negative impacts might differ by SES, as has been shown for losses in school readiness^11^ and language development^10,12^. However, to date no study has examined potential effects of the pandemic on specific social cognition skills, including false-belief understanding.

Here we addressed this gap in the literature by examining potential effects of the pandemic on preschoolers’ false-belief understanding. These data were drawn from a larger study on the relationship between SES and cognitive development. This study took place in California, which had a lengthy stay-at-home order that lasted from March 2020 to June 2021. Data collection for this study began in August 2019 but was paused from March 2020 to September 2021 due to pandemic closures and restrictions on in-person data collection with families. We did not originally set out to study the effects of COVID-19. However, this pause in data collection resulted in an accidental cohort design, enabling us to examine whether false-belief performance differed in children tested before and after COVID-19 lockdowns. Due to the focus of the original study, the sample was socioeconomically diverse. Based on previous evidence that impacts of the pandemic differ by SES^10–12^, we predicted that any cohort differences in false-belief understanding would be larger for children from lower SES households.

## Method

### Participants

Participants were drawn from a larger study on the relationship between socioeconomic status (SES) and cognitive development. 104 children completed at least one testing session. Of this original sample, 8 were excluded from the present study because their parent reported they had a speech delay (3), the parent declined to provide information on family SES (1), the child refused to participate in the language assessment (2), or the child did not have useable data for either elicited-response false-belief task (2) (See Supplementary Information for details on exclusion criteria).

The final sample consisted of 96 children between 3.55 and 5.56 years of age (*M* = 4.65 years, *SD* = 0.60 years). All children were fluent English speakers with no known speech or developmental delays (see Table 1 for demographic information). Children in the pre-pandemic cohort were tested between July 2019 and March 2020; those in the post-pandemic cohort were tested between September 2021 and November 2023. The two cohorts did not differ significantly in age, *t*(94) = 0.80, *p* = .43, parental education, χ^2^(5) = 7.47, *p* = .19, or household income, χ^2^(5) = 6.45, *p* = .17.

**Table 1:** Demographic information.

	Pre-pandemic cohort *N* = 41	Post-pandemic cohort *N* = 55
Age	*M* = 4.71 years, *SD* = 0.57, range: 3.65–5.50	*M* = 4.61 years, *SD* = 0.63, range: 3.55–5.56
Gender	24 female, 17 male	26 female, 29 male
Race		
American Indian/Alaska Native	1	0
Asian	2	4
Black or African-American	0	2
White	22	32
Other race	8	6
More than one race	5	7
N/A	3	4
Ethnicity		
Hispanic or latinx	17	29
Not hispanic or latinx	23	24
N/A	1	2
Parent education		
High school or less	12	6
Associate’s degree	5	8
Bachelor’s degree	12	18
Master’s degree	8	10
PhD/MD	4	13
Household income		
< 20k	2	3
20-40k	8	3
40-60k	8	10
60-80k	7	6
> 80k	16	32
N/A	0	1

N/A indicates the parent declined to provide this information. Parent education reflects the highest degree obtained by either parent.

The children’s names were obtained from a database of parents in Merced County, California who had expressed interest in participating in research studies with their children. Parents were paid $10 for each visit, and each child was given a book for participating. The research was conducted in accordance with the Common Rule guidelines established by the US Office of Human Research protections. The University of California Merced Institutional Review Board approved all procedures (UCM2018-188). All parents provided written informed consent prior to participation.

## Measures and procedure

The larger study involved a battery of tasks completed across two lab visits (*M* time between visits = 5.8 days, *SD* = 4.3 days). Here we examined six of these tasks: three measures of false-belief understanding, two inhibitory-control tasks, and an assessment of children’s language skills. Measures of inhibitory-control and language ability were included because these skills have been shown to correlate with children’s false-belief performance^31,32^.

Each lab visit lasted approximately 1 h. 7 families failed to return for the second visit; these children were retained in the current study because they completed an elicited-response task, an inhibitory-control task, and the language assessment at the first visit. Full details on the breakdown of tasks by visit, exclusion criteria for each task, and missing data for each task can be found in the Supplementary Information.

*Elicited-response tasks* Children completed two widely-used elicited-response false-belief tasks: an unexpected-contents task^33^ and a change-of-location task^34^. Considerable research suggests that children in the United States begin to pass these tasks between 4 and 5 years of age^35^.

In the unexpected-contents task, children were shown a familiar container (crayons box) and asked what they thought was inside (for full procedural details for all tasks, see Supplementary Information). They were then shown that the container actually contained Band-aids. The experimenter then asked the child three questions: what they thought was inside the box when they first saw it (test question 1), what a naïve puppet would think was inside the box (test question 2), and what was actually inside the box (memory question).

In the change-of-location task, children saw a puppet (Piggy) place a toy in one of two containers and then leave. In its absence, a second puppet (Doggy) moved the toy to the other container. The original puppet then returned and children were asked three questions: where Piggy would look for its toy (test question), where Doggy put the toy (memory question 1) and where the toy was now (memory question 2).

For each task, children who answered a memory question incorrectly received a score of 0 for that task (unexpected-contents: *n* = 17; change-of-location: *n *= 5). If children answered the memory questions correctly, they received 1 point for each correct test question for that task. This scoring scheme was determined prior to the onset of data collection based on recommended best practices for hard-to-collect samples^36^. We did not exclude children who failed a memory question due to the difficulty of collecting this sample. This was especially critical here because it is impossible to increase the sample size of the pre-pandemic cohort. Our scoring scheme enabled us to retain as many children as possible in the sample while ensuring children only received credit for a correct false-belief response if they also demonstrated comprehension of the task.

*Low-demand elicited-response task* This task, which was taken directly from Setoh et al.^37^, is designed to impose fewer demands than traditional elicited-response tasks. Several studies conducted prior to the pandemic found that children could pass this task as early as 2.5 years of age^37–39^. Children heard a change-of-location false-belief story accompanied by a picture book. In the story, the main character, Emma, found an apple in one location (basket or box; counterbalanced), placed it in the other location, and then left. In her absence, her brother Ethan found the apple and took it away to an undisclosed location. To reduce processing demands (a) the apple is removed from the scene, reducing the need to inhibit a reality bias, and (b) two practice questions were interleaved with the story to familiarize children with ‘where’ questions and producing the required response (pointing to one of two images). On the final page of the story, children were shown the two locations (sides counterbalanced) and asked where Emma would look for her apple. Pointing to or labeling the false-belief location (i.e. where Emma left her apple) received a score of 1; all other responses received a score of 0.

*Inhibitory-control tasks* Children completed two inhibitory-control tasks: Day/Night^40^ and Grass/Snow^41^. In each task, children had to produce a verbal label that was incongruous with the image they were shown (i.e. saying “day” to a picture of a moon). For each task, the experimenter first introduced the rule and then children received up to 4 practice trials in which they received feedback on their responses. They then received 16 test trials in a semi-randomized order^42^; no feedback was given during the test trials. For each task, we calculated the percentage of test trials in which children produced the correct label. The two inhibitory-control tasks were highly correlated, *r* = .40, *p* < .001; we therefore averaged the two tasks to create a composite inhibitory control score.

*Language assessment* Children completed the Test of Early Language Development, Fourth Edition (TELD-4^43^), a comprehensive measure of spoken language that is normed for children between 3 and 8 years of age. The TELD-4 includes separate subtests for Receptive and Expressive language, which can be combined to create an overall Spoken Language Index. Because two children did not complete the Expressive language subtest (see Supplementary Information), the standardized Receptive language score was used in all models that include language skills as a covariate. However, analyses using the Expressive language score or Spoken Language Index produced the same pattern of significant results.

*Socioeconomic status* We collected two measures of family SES: the highest degree obtained by either parent and the total household income over the past year. These variables were standardized and summed to create a composite SES score. Participants were then divided into higher and lower SES groups via a median split on this composite SES variable. We used a composite SES score, rather than examining education and income separately, because a recent meta-analysis indicated that composite measures of SES yield stronger relationships with false-belief performance^27^.

## Results

All analyses were conducted in R version 4.2.2^44^. All *p-*values in this report are two-tailed. For significant interactions, post-hoc tests were conducted using the *emmeans* package^45^; all such tests were corrected for multiple comparisons. For each model, preliminary analyses were conducted to examine potential effects of child sex, age, or language skills, visit number, and task order. Where significant, these factors were controlled for in the final model. Full model results are provided in the Supplementary Information.

Children’s responses to the elicited-response test questions were analyzed with a generalized linear model with cohort (pre-pandemic, post-pandemic), SES group (lower, higher) and their interactions as between-subjects factors and child age and Receptive language score as covariates. The model was specified with a binomial distribution and a logit link function because the outcome for each test question was binary. We report *p*-values obtained via likelihood ratio tests. Inhibitory control was not included in this model as a covariate because preliminary analyses indicated no significant relationship between inhibitory control and elicited-response performance, *p* = .42.

**Fig. 1:**
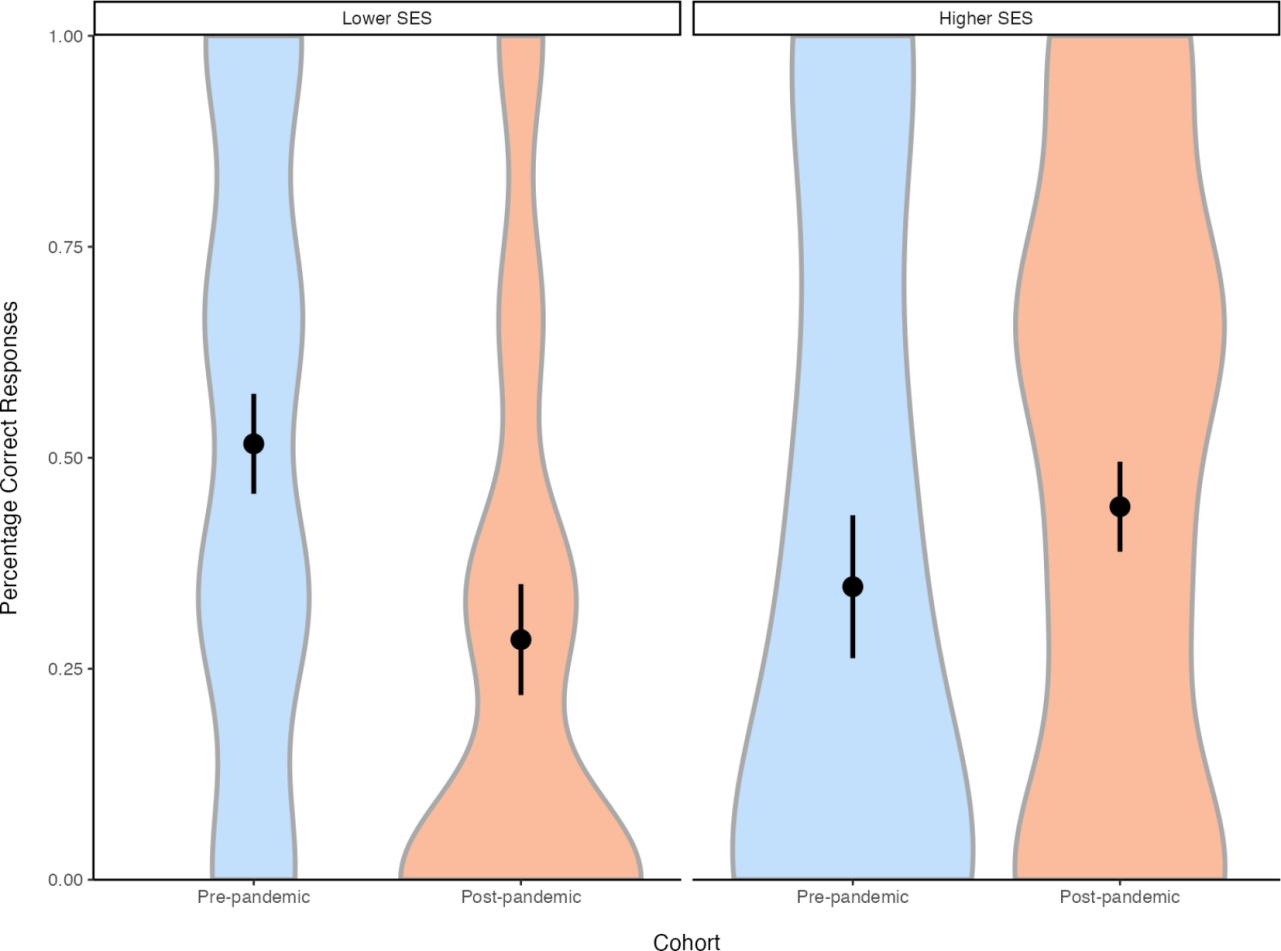
Violin plot of performance on the elicited-response false-belief tasks, separately by cohort and SES group. The y-axis represents the percentage of false-belief test questions (out of 3) answered correctly. Within each violin, the black dot indicates the mean and vertical lines represent one standard error of the mean.

The model revealed a significant effect of cohort, *β* = 0.99, *SE* = 0.40, χ^2^(1) = 6.36, *p* = .012. Children tested pre-pandemic performed significantly better than children tested post-pandemic. There was also a significant interaction of cohort and SES group, *β* = −1.39, *SE* = 0.59, χ^2^(1) = 5.72, *p* = .017 (see Fig. 1). Tukey corrected comparisons showed that in the lower SES group, children tested post-pandemic performed significantly worse than children tested pre-pandemic, *z* = −2.47, *p* = .014. In contrast, in the higher SES group, the pre- and post-pandemic cohorts did not differ significantly from one another, *z* = 0.93, *p* = .35.

**Fig. 2:**
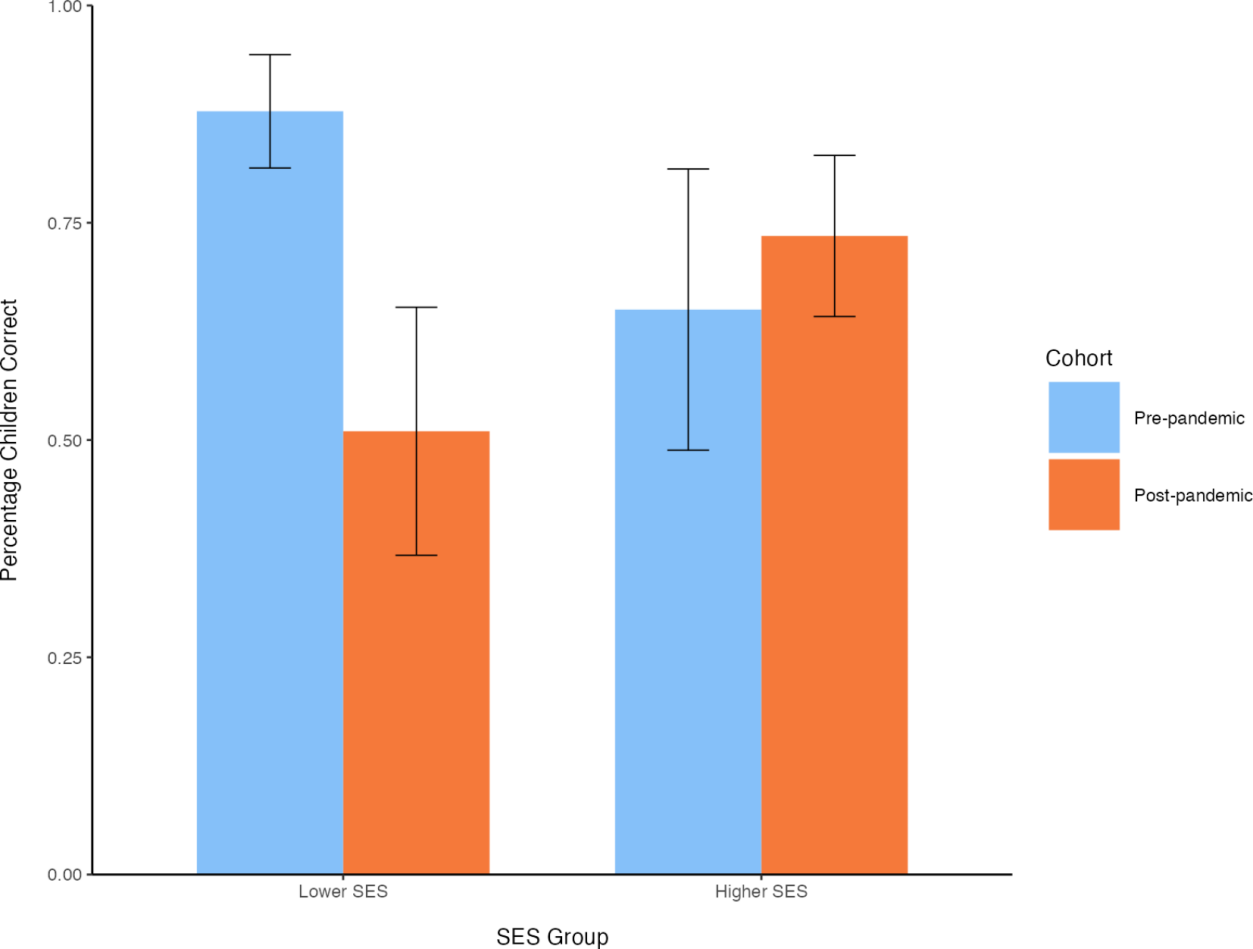
Performance on the low-demand elicited-response task, separately by cohort and SES group. The y-axis represents the percentage of children who answered the test question correctly. Error bars represent one standard error of the mean.

Children’s performance on the low-demand elicited-response task was also analyzed with a generalized linear model specified with a binomial distribution and logit link function. The model included cohort, SES group, and their interactions as between-subjects effects, and child age, child sex, and visit number as covariates. This model revealed a significant effect of cohort, *β* = 1.94, *SE* = 0.84, χ^2^(1) = 5.83, *p *= .016; children in the pre-pandemic cohort again performed significantly better than those in the post-pandemic cohort. Note that in the pre-pandemic cohort, 79% of the children answered the test question correctly, which is comparable to performance in previous studies using this task with 2.5-year-old children^37–39^. There was also a significant interaction of cohort and SES group, *β* = −2.34, *SE* = 1.19, χ^2^(1) = 4.00, *p* = .046 (see Fig. 2). Tukey corrected comparisons again showed that in the lower SES group, children performed significantly worse post-pandemic, *z* = −2.31, *p* = .021. In the higher SES group, the pre- and post-pandemic cohorts did not differ, *z* = 0.47, *p* = .64.

These findings indicate that children tested after the pandemic demonstrated deficits in false-belief performance relative to those tested before the pandemic. However, it is possible that these performance differences reflect deficits in other cognitive skills, rather than difficulties with false-belief understanding per se. We addressed this possibility in several ways.

First, poorer performance on the elicited-response tasks could stem from difficulty with the memory questions, as children who answered these questions incorrectly received a 0 for the task. We therefore examined whether children tested after the pandemic were more likely to respond incorrectly on the memory questions in the elicited-response tasks compared to those tested before the pandemic. Responses to the memory questions were analyzed with a generalized linear model specified with a binomial distribution and a logit link function, cohort (pre-pandemic, post-pandemic), SES group (lower, higher) and their interactions as between-subjects factors, and child age and Receptive language score as covariates. This model revealed a significant interaction of cohort and SES group, *β* = −2.18, *SE* = 0.99, χ^2^(1) = 4.88, *p* = .027. Tukey corrected comparisons showed that in the higher SES group, children tested post-pandemic performed significantly better on the memory questions than those tested pre-pandemic, *z* = 2.35, *p* = .019. In the lower SES group, the pre- and post-pandemic cohorts did not differ, *z* = − 0.72, *p* = .47. Whereas performance on the false-belief test questions was lower post-pandemic, performance on the memory questions was equal (lower SES) or better (higher SES) post pandemic. This suggests that the effects we observed for false-belief performance were not driven by post-pandemic deficits in memory performance.

Second, we examined whether the pre- and post-pandemic cohorts differed in their inhibitory control or language skills. Inhibitory control scores were analyzed with a generalized linear model with a binomial distribution and a logit link function, cohort (pre-pandemic, post-pandemic), SES group (low, high) and their interactions as between-subjects factors, and child age as a covariate. There were no significant effects or interactions involving cohort or SES group, all *p*s > 0.31. Similarly, each language score (Receptive, Expressive, Spoken Language Index) was analyzed with a linear regression with cohort (pre-pandemic, post-pandemic), SES group (low, high), and their interactions as between-subjects effects. These models did not reveal any significant effects, all *p*s > 0.28. These results suggest that the difficulties children exhibited post-pandemic were specific to false-belief performance.

## Discussion

The COVID-19 pandemic and ensuing lockdowns dramatically changed the daily lives of children and their families. The present study was the first to examine the potential impact of these changes on the development of social cognition in early childhood. We found that, relative to the pre-pandemic cohort, 3.5- to 5.5-year-old children tested after the pandemic performed significantly worse on several measures of false-belief understanding. The two cohorts did not differ in their inhibitory control or language ability, suggesting that the deficits in the post-pandemic cohort were specific to their social cognition skills.

These findings add to a growing body of evidence that the COVID-19 pandemic negatively impacted many aspects of child development^1,8–11,14,15^. Our work extends these findings to early social cognition skills, providing the first evidence of post-pandemic deficits in children’s false-belief understanding. The fact that deficits emerged for the low-demand task was particularly striking, given that several studies conducted prior to the pandemic found that children were able to pass this task at 2.5 years of age^37–39^, several years younger than the present sample. False-belief understanding has long been viewed as a critical component of social cognition skills and it is correlated with many positive developmental outcomes. Thus, the delays we observed in the post-pandemic cohort could have consequences not only for children’s social cognition abilities, but also for other domains including prosocial behavior^20^, relationships with peers^19^, and academic performance^18^.

Moreover, the difference between the two cohorts was greater for children from lower SES backgrounds. This is consistent with other recent evidence that the pandemic had a greater impact on lower SES households, including greater losses in school readiness in preschool children^11^ and greater learning losses for school-aged children^1^. Previous research suggests that lower SES children are at greater risk for delays in several developmental outcomes^46–48^, including false-belief understanding^27^. Our work contributes to a growing body of work suggesting that the pandemic exacerbated such disparities, contributing to greater socioeconomic inequality in development.

Why did children in the post-pandemic cohort exhibit poorer false-belief performance and why was this difference larger for lower SES children? Although we cannot directly address this question with the present data, we speculate that these effects resulted from shifts in both the number and nature of social interactions that children had during the pandemic. The development of social cognition skills is linked to children’s social experiences and social contexts^27,49,50^. For instance, positive interactions with peers supports improvements in false-belief reasoning^51^. Pandemic lockdowns likely reduced the opportunity for interactions with peers due to closures of preschools, daycares, and other public spaces, as well as reductions in informal gatherings like playdates due to social distancing policies. Similarly, exposure to and use of mental-state language (e.g., words such as *think*,* want*,* happy*) is positively associated with social cognition skills in infants, toddlers, and preschoolers^27–30,52^. Evidence suggests that parents who are under greater strain talk less to their children^53–55^ and produce fewer questions that reference mental states^56^. Thus, the negative impacts of the pandemic on parents’ mental health^6,7,57^, which were greater in lower SES homes, could have impeded parents’ ability to engage in parent-child interactions that would foster social cognition skills. Finally, it is also possible that conversations about mental states were replaced by other activities such as passive screen time^10,15^, which has been linked to poorer false-belief understanding^58^. Further research on how the pandemic affected young children’s social context and parent-child interactions is needed to test these possibilities.

Unlike several other recent studies that identified negative impacts of the pandemic on infant’s vocabulary development^10,12^ and preschooler’s language skills^14^, we did not find any cohort differences in receptive, expressive, or overall language ability. This difference could stem from the fact that we only collected language data at a single time point. In contrast, Bergmann et al.^10^ and Kartushina et al.^12^ obtained vocabulary data for 8–36-month-old infants at two time points and found that differences in screentime during lockdown were related to differences in changes in vocabulary size over time. Because the present study was cross-sectional, we cannot examine potential differences in the growth of children’s language skills across the two cohorts. Similarly, we also could not control for pre-pandemic skill levels, as was done by Sato et al.^14^. It is possible that if we were able to examine changes in children’s language skills over time, controlling for earlier levels of performance, that cohort differences in language abilities might emerge.

The present study has several limitations. First, we do not have detailed information on the day-to-day interactions of these families, especially during COVID lockdowns, and thus we cannot directly test potential mechanisms for the cohort effects we observed. Second, as previously discussed, this study was cross-sectional, and thus we could not directly compare pre- and post-pandemic performance within individual children. This also raises the possibility that the poorer performance in the post-pandemic cohort was due to sample differences, rather than the impact of the pandemic. Although we cannot directly rule out this possibility, we think it is unlikely because the pre- and post-pandemic cohorts did not differ on any of the other variables that we measured: parent education, household income, age, language ability, or inhibitory control skills. We also found no evidence that children in the post-pandemic cohort performed worse on the memory questions in our tasks. This suggests that the two samples were generally comparable. We therefore think it is more plausible that the poorer social cognition skills in the post-pandemic cohort reflect influences of the pandemic rather than sampling differences.

Despite these limitations, our study makes several important contributions to the literature. It is the first study to examine potential impacts of the pandemic on social cognition skills and on false-belief understanding specifically. We also focused on impacts of the pandemic on early childhood, which has received relatively less attention than the school years (5 years and up). The finding that children in the post-pandemic cohort exhibited poorer social cognition skills has both practical and methodological implications. Our findings suggest a need to provide additional support for children’s social-cognitive development post-pandemic. It may be especially important to provide this support to children from lower SES backgrounds in order to prevent widening socioeconomic inequalities in early development. Methodologically, issues of replicability have recently received considerable attention both in the broader field of psychology^59^ and in work on children’s false-belief understanding in particular^60,61^. Our results suggest that comparisons of studies on false-belief understanding conducted pre- and post-pandemic should be interpreted with caution, as poorer performance post-pandemic could reflect a cohort effect rather than non-replication.

## Electronic supplementary material

Below is the link to the electronic supplementary material.

Supplementary Material 1

## Data Availability

The data and code necessary to reproduce the results in this article (and its Supplementary Information files) are available on OSF at: https://osf.io/9yu2w/?view_only=4bf7c4d60419473887edd48290739516.
