# Supplementary material for: The COVID-19 pandemic and social cognitive outcomes in early childhood
Source: Sci Rep. 2024 Nov 22;14:28939. doi: 10.1038/s41598-024-80532-w (PMC11584632; doi:10.1038/s41598-024-80532-w)
Supplement: Supplementary file 1 — Supplementary Material 1 [file 41598_2024_80532_MOESM1_ESM.docx]

Supplementary Information

**Apparatus and Procedure**

Children sat at a table across from an experimenter; their parent sat in a chair behind them. Parents completed questionnaires during the tasks and were instructed to remain quiet and neutral. On the table sat a wooden storybook apparatus (56 × 53 cm; inclined at a 70**°** angle) that was used for the low-demand elicited-response task. A camera behind the apparatus captured the child’s face. A second camera above and behind the child captured the stimuli and the child’s pointing responses.

At each visit, children completed three tasks: an elicited-response task (unexpected-contents or change-of-location), an inhibitory-control task (Day/Night or Grass/Snow), and a storybook task (low-demand elicited-response task or an unrelated task not analyzed here). The tasks completed at each visit and the order of tasks within visit were counterbalanced across children (see Table S1). The TELD-4^1^ was completed at the end of visit 1, unless children were fatigued and uncooperative, in which case it was administered at visit 2.

Table S1

*Distribution of Tasks Across Visits*

|  | Visit 1 | Visit 2 | Missing |
| --- | --- | --- | --- |
| Unexpected-contents task | 48 | 43 | 5 |
| Change-of-location task | 48 | 42 | 6 |
| Low-demand elicited-response task | 49 | 40 | 7 |
| Day/Night | 46 | 41 | 9 |
| Grass/Snow | 45 | 45 | 6 |
| TELD-4 Receptive | 91 | 5 | 0 |
| TELD-4 Expressive | 90 | 4 | 2 |

**Unexpected-contents task**. In the unexpected-contents task^2^ the experimenter held up a crayons box and asked, “What do you think is in this box?” If children did not answer, this prompt was repeated up to 3 additional times. All children responded before proceeding. Next, the experimenter opened the box and showed the contents (Band-Aids) to the child. Children were then asked, “What are they?” If children responded correctly (Band-aids or bandages), the experimenter confirmed the response saying, “Yes, that’s right. They are Band-aids.” If children did not respond, the experimenter asked up to 3 additional times. If the children still did not respond, or if they responded incorrectly, the experimenter provided the correct answer: “They are Band-aids right?” The experimenter then returned the Band-aids to the box and closed it so that the contents were not visible.

The experimenter then asked the children two questions: “When you first saw this box, what did you think was inside?” (test question 1) and “Do you remember what is actually inside the box?” (memory question). Next, the experimenter retrieved a bear puppet from a closed box under the table and said, “Look! This is my friend, Bear. Bear hasn’t seen inside this box. What will Bear think is inside this box?” (test question 2). For the test and memory questions, if the children did not respond to the initial question, the experimenter asked up to 3 additional times before moving on to the next question. No praise or feedback was given for children’s responses to these questions.

**Change-of-location task.** In the change-of-location task^3^, two closed containers (a box and a lidded bucket) were placed on the table to the right and left of the children, respectively. The experimenter brought out a pig puppet and a dog puppet and introduced them saying, “This is Piggy and this is Doggy.” The experimenter then showed the children that Piggy had a small, red teddy bear. Piggy placed the teddy bear in one of the two containers (counterbalanced across children). While performing this action, the experimenter said, “Piggy puts the teddy bear in the [container] to keep it safe. Then Piggy goes outside to play.” The experimenter then hid Piggy from view behind the storybook apparatus. Doggy then removed the teddy bear from the original hiding location and placed it in the other container, stating, “While Piggy is gone, Doggy takes the teddy bear out of the [original container]. Doggy puts the teddy bear in the [other container] and then he leaves.” The experimenter then placed Doggy out of view beneath the table. At this point, the experimenter retrieved Piggy, held Piggy centered between the two containers, and said, “Piggy comes back inside. Piggy wants to play with his teddy bear.” The children were then asked three questions: “Where will Piggy look for his teddy bear?” (test question), “Where did Doggy put the teddy bear?” (memory question 1), and “Where is the teddy bear?” (memory question 2). For the test and memory questions, if the children did not respond to the initial question, the experimenter asked up to 3 additional times before moving on to the next question. No praise or feedback was given for children’s responses to these questions.

**Low-demand elicited-response task.** This task was taken directly from Setoh et al.^4^ Children heard a change-of-location false-belief story accompanied by images on the storybook apparatus. 9 storybook pages were attached to the top of the apparatus with binder rings. Each page (56 × 28 cm) consisted of a clear plastic photo sheet with white paper backing; one or two color photos (20 × 25 cm) were affixed to the sheet. Single photos were centered, and double photos were 4.5 cm apart.

The story introduced Emma (story trial 1; see Setoh et al.^4^ for images and script), who found an apple in one of two containers (story trial 2; container counterbalanced across children). Emma placed her apple in the other container (story trial 3) and then went outside to play with her ball (story trial 4). While she was gone, her brother Ethan found the apple and took it away to an undisclosed location (story trial 5). This removal of the apple from the scene was intended to reduce inhibitory demands in the test trial^4^. Emma then returned to look for her apple (story trial 6). In the test trial, children saw pictures of the two containers (sides counterbalanced across children) and were asked, “Where will Emma look for her apple?”

The task included two practice trials in which children were shown two pictures and asked a ‘where’ question. After story trial 2, children saw an apple and a banana and were asked, “Where is Emma’s apple?” After story trial 4, they saw a frisbee and a ball and were asked, “Where is Emma’s ball?” These trials were intended to reduce the response-generation demands of the test trial by giving children practice interpreting and responding to ‘where’ questions by pointing to one of two images.

The experimenter stood behind the storybook apparatus across from the child. The pages of the book began face down behind the apparatus. On each story trial, the experimenter turned a page towards the child, recited a line of the story, and then paused briefly, looking naturally between the page and the child. In each practice trial, the experimenter turned the page, asked the practice question, and then paused for up to 5 seconds. If the child responded correctly (164/176 trials), the experimenter praised the child and continued the story. If the child did not respond (6/176 trials), the experimenter asked up to 2 additional times (all children responded by the third prompt). If children responded incorrectly (6/176 trials), the experimenter prompted the child again, ensuring the child responded correctly before proceeding. Averaged across practice trials, children required 1.04 prompts (*SD* = .19).

In the test trial, the experimenter turned the page, asked the test question, and paused for up to 5 seconds. If the child did not respond, the experimenter asked up to four additional times. On average, children required 1.12 prompts (*SD* = .52). Throughout the practice and test trials, the experimenter looked directly at the children to ensure that they (1) would interpret the question as a direct question^5^ and (2) could not use the experimenter’s gaze as a cue for where to point.

**Inhibitory control tasks**. Children completed two inhibitory control tasks: Day/Night^6^ and a modified version of Grass/Snow^7^. Both tasks required children to produce a label that was incongruent with the picture they were shown (e.g., “Night” for a picture of a sun).

In the Day/Night task, the experimenter first asked children when they see the sun and moon and ensured they said “day” and “night,” respectively. The experimenter then held up a picture of a sun and explained that when shown this image, children should say “Night.” The children were asked to repeat “Night” before proceeding. This was repeated with an image of a moon and stars, for which children were told to say “Day.” After explaining the rules, all children received two practice trials, one with each image: the experimenter held up the image and asked, “What do you say for this one?” If children responded correctly, the experimenter praised their responses and moved on to the test trials. If children did not respond or answered either practice trial incorrectly, the experimenter corrected them and reminded them of the rules, then administered an additional set of practice trials. If children again failed to answer both practice trials correctly, the experimenter repeated the rules again and administered a third set of practice trials. The experimenter then proceeded to the test trials. The majority of children (*N* = 53) completed one set of practice trials, 20 received two sets of practice trials, and 13 received all three sets of practice trials.

Children received 14 test trials in a fix random order. In each trial, the experimenter held up one of the two images and asked children what they said for that image. If children did not respond, the experimenter repeated the prompt up to 3 additional times before moving on to the next test trial. No praise or feedback was given during the test trials.

The procedure for Grass/Snow was identical except that the sun and moon images were replaced by green and white sheets of construction paper and children had to say ‘grass’ for the white paper and ‘snow’ for the green paper. At the start of the task, the experimenter asked the children what color grass and snow were and ensured they said ‘green’ and ‘white’ respectively. The experimenter then proceeded as described above. The majority of children (*N* = 63) completed one set of practice trials, 22 received two sets of practice trials, and 5 received all three sets of practice trials.

Note that in the original Grass/Snow task, children were shown both the green and white images and the same time and asked to point to the green and white when the experimenter said ‘snow’ and ‘grass,’ respectively^7^. Given children’s performance in previous studies^7^, it seemed likely that much of the current sample would perform at ceiling in this pointing version of the task. We thus adapted the task to align with the procedure for Day/Night, requiring children provide an opposing label for a given image.

**Exclusions and missing data**

Three children in the original sample were excluded because their parent reported that they had a diagnosed speech delay. Of the remaining sample, children were included in the present study if (a) their parent provided at least one of the SES indicators (education or household income) and (b) the child had useable data for at least one elicited-response task and the TELD-4 Receptive subtest. Based on these criteria, 5 additional children were excluded because the parent declined to provide information on both household income and parental education (1), the child refused to complete either portion of the TELD-4 (2), or the child did not have useable data for either elicited-response task (2). With regards to these last 2 children, both children completed the unexpected-contents task on visit 1 but were eliminated due to experimenter error and then failed to return for visit 2.

The next section provides details on missing data for the final sample of 96 children and how these missing data were handled in analyses.

**Elicited-response tasks**. For the unexpected-contents task, 1 child was excluded due to experimenter error (failure to ask all test and memory questions) and 4 children did not complete the task because they did not return for visit 2. For the change-of-location task, 3 children were excluded due to experimenter error (failure to close container so toy was visible to Piggy during the test and memory questions) and 3 did not complete the task because they did not return for visit 2. In order to include children who had completed only one of the two tasks, elicited-response performance was analyzed as the number of test questions answered correctly as a function of the number of questions administered.

**Low-demand elicited-response task**. Two children did not complete the task because they failed to return for the second visit. Five additional children were excluded due to experimenter errors when reading the script or asking a practice question (e.g., calling the ball an apple, reciting lines out of order).

**Inhibitory-control tasks**. For Day/Night, 1 child refused to complete the activity, 3 were excluded for experimenter error (failure to properly administer the practice trials), and 5 did not complete the task because they failed to return for the second visit. For Grass/Snow, 4 children were excluded due to experimenter error (failure to properly administer the practice trials), and 2 did not complete the task because they did not complete their second visit. In order to include children who completed only one of the two tasks, inhibitory-control performance was analyzed as the number of test questions answered correctly out of the number of test trials administered.

**TELD-4**. Two children completed the TELD-4 Receptive subtest and then refused to continue with the Expressive subtest. The Receptive score was therefore used in all analyses involving the TELD-4.

**Full model results**

Table S2

*Generalized Linear Model on Elicited-Response Scores*

| Effect | *β* | *SE* | χ^2^ | *p-*value |
| --- | --- | --- | --- | --- |
| **Cohort (pre/post-pandemic)** | **.99** | **.40** | **6.36** | **.012*** |
| SES group | .69 | .39 | 3.26 | .071^ |
| **Receptive Language** | **.07** | **.02** | **18.34** | **<.001***** |
| **Child age** | **.08** | **.02** | **16.49** | **<.001***** |
| **Cohort*SES group** | **-1.39** | **.59** | **5.72** | **.017*** |

Table S3

*Generalized Linear Model on Test Question Responses in Low-Demand Elicited-Response Task*

| Effect | *β* | *SE* | χ^2^ | *p-*value |
| --- | --- | --- | --- | --- |
| **Cohort (pre/post-pandemic)** | **1.94** | **.84** | **5.83** | **.016*** |
| SES group | .98 | .75 | 1.76 | .185 |
| **Sex** | **-1.39** | **.61** | **5.76** | **.016*** |
| **Visit** | **-1.78** | **.45** | **20.55** | **<.001***** |
| **Cohort*SES group** | **-2.34** | **1.19** | **4.00** | **.046*** |

Table S4

*Generalized Linear Model on Memory Question Responses in Elicited-Response Tasks*

| Effect | *β* | *SE* | χ^2^ | *p-*value |
| --- | --- | --- | --- | --- |
| Cohort (pre/post-pandemic) | .49 | .68 | .52 | .471 |
| SES group | .86 | .71 | 1.50 | .221 |
| **Receptive Language** | **.05** | **.03** | **3.94** | **.047*** |
| Child age | .04 | .03 | 1.36 | .243 |
| **Cohort*SES group** | **-2.18** | **.99** | **4.88** | **.027*** |

Table S5

*Generalized Linear Model on Inhibitory Control Scores*

| Effect | *β* | *SE* | χ^2^ | *p-*value |
| --- | --- | --- | --- | --- |
| Cohort (pre/post-pandemic) | .04 | .14 | .06 | .800 |
| SES group | -.03 | .13 | .05 | .818 |
| **Child age** | **.04** | **.007** | **32.28** | **<.001***** |
| Cohort*SES group | .21 | .21 | 1.00 | .315 |

Table S6

*Linear Regression on Receptive Language Scores*

| Effect | *β* | *SE* | *t* | *p-*value |
| --- | --- | --- | --- | --- |
| Cohort (pre/post-pandemic) | .12 | .30 | .395 | .694 |
| SES group | .24 | .29 | .858 | .393 |
| Cohort*SES group | -.13 | .44 | -.292 | .771 |

Table S7

*Linear Regression on Expressive Language Scores*

| Effect | *β* | *SE* | *t* | *p-*value |
| --- | --- | --- | --- | --- |
| Cohort (pre/post-pandemic) | .32 | .30 | 1.066 | .289 |
| SES group | .24 | .28 | .843 | .401 |
| Cohort*SES group | .006 | .44 | .014 | .989 |

Table S8

*Linear Regression on Spoken Language Index Scores*

| Effect | *β* | *SE* | *t* | *p-*value |
| --- | --- | --- | --- | --- |
| Cohort (pre/post-pandemic) | .31 | .30 | 1.05 | .296 |
| SES group | .24 | .28 | .862 | .391 |
| Cohort*SES group | -.03 | .44 | -.074 | .941 |

**References**

1. Hresko, W.P., Reid, D.K., & Hammill, D.D. *TELD-4: Test of Early Language Development*. (Pro-Ed, 2017).
2. Gopnik, A., & Astington, J. W. Children’s understanding of representational change and its relation to the understanding of false belief and the appearance–reality distinction. *Child Dev.* **59,** 26–37 (1988). <https://doi.org/10.2307/1130386>.
3. Baron-Cohen, S., Leslie, A. M., & Frith, U. Does the autistic child have a “theory of mind*”? Cognition,* **21,** 37-46 (1985). <https://doi.org/10.1016/0010-0277(85)90022-8>.
4. Setoh, P., Scott, R. M., & Baillargeon, R. Two-and-a-half-year-olds succeed at a traditional false-belief task with reduced processing demands. *Proc. Nat. Acad. Sci. USA* **113,** 13360-13365 (2016). <https://doi.org/10.1073/pnas.1609203113>.
5. He, Z., Bolz, M., & Baillargeon, R. 2.5‐year‐olds succeed at a verbal anticipatory‐looking false‐belief task. *Br. J. Dev. Psychol.* **30**, 14-29 (2012). <https://doi.org/10.1111/j.2044-835X.2011.02070.x>.
6. Gerstadt, C. L., Hong, Y. J., & Diamond, A. The relationship between cognition and action: performance of children 312–7 years old on a stroop-like day-night test. *Cognition,* **53,** 129-153 (1994). <https://doi.org/10.1016/0010-0277(94)90068-X>.
7. Carlson, S. M., & Moses, L. J. Individual differences in inhibitory control and children's theory of mind. *Child Dev.* **72,** 1032-1053 (2001). <https://doi.org/10.1111/1467-8624.00333>.
